# Emergence of tigecycline-resistant *Raoultella ornithinolytica* with *tet*(X)-carrying plasmid from swine wastewater in China

**DOI:** 10.3389/fmicb.2025.1642708

**Published:** 2025-09-16

**Authors:** Qing Jia, Haobo Jin, Xi Jin, Xinlong Zhu, Chaoyue Cui

**Affiliations:** ^1^Laboratory Animal Centre, Wenzhou Medical University, Wenzhou, Zhejiang, China; ^2^Department of Clinical Laboratory, The Second Affiliated Hospital of Wenzhou Medical University, Wenzhou, Zhejiang, China

**Keywords:** *: tet*(X), *Raoultella ornithinolytica*, IncFII plasmid, *Klebsiella pneumoniae*, tigecycline

## Abstract

**Background:**

The increasing prevalence of antibiotic resistance genes (ARGs), such as the plasmid-mediated tigecycline-modifying enzyme *tet*(X), significantly hinders the treatment of infectious diseases in humans and animals. Livestock wastewater contributes to the transmission of these ARGs.

**Methods:**

Between June 2023 and December 2024, 140 wastewater samples from 15 swine farms in Shandong, China, were screened for *tet*(X)-positive strains using PCR and 16S rRNA sequencing. *Raoultella ornithinolytica* SD8 was assessed for antimicrobial susceptibility, plasmid stability, conjugation, fitness cost, and pathogenicity in a BALB/c mouse model. Furthermore, this strain was subjected to whole-genome sequencing.

**Results:**

*tet*(X4) was found to be located on a 78,159 bp IncFII(pCRY)-like plasmid (pSD8-1-2) in *R. ornithinolytica* SD8-1, exhibiting high stability (92% retention after 20 days) and conjugative transfer to *Escherichia coli* C600 and *bla*_*NDM*_-producing E218 at frequencies of 1.6 × 10^–5^ and 4.3 × 10^–6^, respectively, with minimal fitness cost. Studies in mice showed that *R. ornithinolytica* SD8-1 caused severe organ damage. pSD8-1-2 led to tigecycline treatment failure, unlike the plasmid-cured strain. Database analysis identified pSD8-1-2-like plasmids or fragments were identified predominantly in *Klebsiella pneumoniae*, indicating a potential risk of dissemination.

**Conclusion:**

The *tet*(X4)-carrying plasmid pSD8-1-2 in *R. ornithinolytica* SD8-1 exhibits high stability and cross-species transferability, exacerbating tigecycline resistance and treatment failure. Based on the “One Health” concept, the spread of this plasmid into humans in clinical settings should be closely monitored.

## Introduction

The rise in antibiotic resistance presents a significant threat to human health. By 2035, resistance to last-resort antibiotics is projected to be 2.1 times higher than that in 2005. Currently, around 7.7 million people die each year from bacterial infections, of which antibiotic-resistant bacteria cause 4.95 million deaths (Okeke et al., 2024). Livestock farms, a major sector for antibiotic application, are critical sites for the evolution and spread of antibiotic resistance genes (ARGs) (Gupta et al., 2019). Due to their high abundance in farm manure, ARGs can spread into the environment through wastewater or be transferred to crops via fertilization and irrigation. Contamination of agricultural products with livestock feces or wastewater may introduce resistant or pathogenic bacteria into the food chain, posing potential risks to humans (Chu et al., 2024; Holvoet et al., 2013; Onalenna and Rahube, 2022). Improper use of antibiotics in the livestock industry most likely contributed to the emergence of genes, such as mobilized colistin resistance (*mcr*) and *tet*(X), which cause resistance to last-resort antibiotics, colistin and tigecycline, respectively (Aminov, 2021). After China officially banned the use of colistin as a prophylactic growth-promoting feed additive in 2017, the detection rate of *mcr* in all ecological niches, including clinical settings, dropped sharply, providing direct evidence for this view (Sun et al., 2023; Wang et al., 2020).

Since the discovery of novel *tet*(X) variants in China in 2019, it has attracted attention due to its association with tigecycline resistance (He et al., 2019; Sun et al., 2019; Zhang et al., 2020). Currently, *tet*(X)-positive strains are widely distributed across diverse sources, including food, meat, vegetables, wild birds, and human clinical and intestinal samples (Cui et al., 2020, 2022; Fan et al., 2024; Fang et al., 2020). However, they are predominantly detected in farms and surrounding environments. Among its variants, *tet*(X4) stands out because it has a clear preference for Enterobacteriaceae, mainly Escherichia coli, and has a higher activity. In addition to *E. coli*, *tet*(X) variants have been sporadically detected in *K. pneumoniae* and Salmonella (Liu X. et al., 2024; Yue et al., 2024; Zhang et al., 2024), but fortunately, its prevalence is not widespread in these species. *tet*(X4) is usually present on plasmids such as IncX1, IncQ, IncFII, IncFIB and mixed plasmids, some of which have a broad host range, facilitating its spread (Fang et al., 2020; Li et al., 2020).

*R. ornithinolytica* is a common bacterium primarily found in soil and aquatic environments, with additional reports in insects and fish. Although it was originally classified in the genus *Klebsiella*, advances in molecular techniques resulted in its re-classification into a newly formed genus *Raoultella* in 2001 (Drancourt et al., 2001). It can cause histamine poisoning, due to its ability to convert histidine into histamine (Hajjar et al., 2020). Although *R. ornithinolytica* is relatively rare, its infection rate has been steadily rising. *R. ornithinolytica* causes infections in various organs, including the respiratory tract, bloodstream, and soft tissues in the urinary tract (Hajjar et al., 2020; Jones et al., 2024). Moreover, this species often exhibits multidrug resistance (MDR) similar to that of *K. pneumoniae*, with reports of key resistance genes such as *bla*_*KPC–2*_, *mcr* (Xedzro et al., 2023), and *tmexCD-toprJ* (Zhu et al., 2023), posing a substantial threat to patients with underlying diseases or compromised immunity.

In this study, a *tet*(X4)-producing *R. ornithinolytica* strain was identified during routine large-scale wastewater surveillance at pig farms. Given the close phylogenetic relationship between *R. ornithinolytica* and the clinically significant pathogen *K. pneumoniae*, the antimicrobial resistance, pathogenicity, and plasmid characteristics of this strain were comprehensively analyzed.

## Materials and methods

### Sample collection and identification of *tet*(X)-positive strains

From June 2023 to December 2024, we collected a total of 140 non-repetitive sewage samples during the monitoring of sewage resistance in swine farms in Shandong Province. A total of 140 sewage samples were obtained from 15 different farms distributed across five cities, with each farm contributing between 7 and 14 samples. A total of 50 μl of sewage sample was transferred to 800 μl of LB broth and cultured overnight at 37 degrees with shaking. Then, overnight cultures were inoculated onto LB agar plates containing 2 mg/L tigecycline using an inoculation loop. Detection of common tigecycline resistance genes using PCR in resistant colonies. 16S DNA and Sanger sequencing were used for strain identification.

### Antimicrobial susceptibility testing

Antimicrobial susceptibility of strains was determined by agar dilution method according to Clinical and Laboratory Standards Institute (CLSI) guidelines (Humphries et al., 2018). A panel of 14 antimicrobial drugs was tested: amikacin (AMK), gentamicin (GEN), fosfomycin (FOS), rifampicin (RIF), tetracycline (TET), ampicillin (AMP), cefoxitin (FOX), cefotaxime (CTX), meropenem (MEM), trimethoprim-sulfamethoxazole (SXT), and florfenicol (FFC). The MICs of tigecycline (TGC) and colistin (CS) were examined by the broth microdilution method. The breakpoints for tigecycline and colistin were interpreted as resistant at > 0.5 and > 2 mg/L, respectively, according to the European Committee on Antimicrobial Susceptibility Testing (European Committee on Antimicrobial Susceptibility Testing [EUCAST], 2025). *E. coli* ATCC 25922 served as the quality control strain.

### Conjugation experiments

To verify the transferability of the pSD8-1-2 plasmid, filter mating experiment was performed using the *E. coli* C600 and the clinical isolates of meropenem-resistant *K. pneumoniae* K251 and K242 as recipient strains. The donor strain and the recipient strain were mixed in a ratio of 1:3 and inoculated into an antibiotic-free LB agar with a 0.22 μm pore size sterile filter membrane. Subsequently, the LB agar medium was incubated at 37 °C for 24 h. Binding compounds were screened on two-drug plates, with TGC (4 mg/L) and streptomycin (2,000 mg/L) added for *E. coli* C600, and TGC (4 mg/L) and MEM (4 mg/L) added for clinical *K. pneumoniae*. PCR and Sanger sequencing were used to confirm whether the transconjugant emerged. Conjugation frequency was calculated as the ratio of transconjugant colony-forming units (CFUs) to recipient CFUs. Briefly, after incubation, cells were collected, serially diluted, and plated on double-antibiotic plates to enumerate transconjugants and on recipient-selective plates to enumerate recipients.

### Plasmid stability

The stability of pSD8-1-2 was investigated by conventional plate count method. Briefly, a single colony of freshly cultured SD8-1 was inoculated into 4 ml of antibiotic-free LB broth and cultured at 37 °C with shaking. The culture was then subcultured in antibiotic-free LB broth at a ratio of 1:1000, subcultured every 12 h, twice a day, for 25 consecutive days. The culture was taken every day, serially diluted, and then inoculated onto antibiotic-free LB agar plates. Following overnight incubation, 50 colonies were randomly selected for PCR amplification of the *tet*(X4) gene. The plasmid retention rate was calculated as the number of *tet*(X4)-positive colonies divided by the total number of colonies.

### Growth curve measurement and statistical analysis

Single colonies of all strains to be tested were selected and inoculated into LB broth, and cultured overnight at 37 °C and 200 rpm with shaking. After adjusting the overnight culture to the 0.5 McFarland turbidity standard, it was diluted 1:100 in LB broth. The optical density was measured at a wavelength of 600 nm (OD_600_) using a microplate reader, recorded every hour, and bacterial growth was continuously monitored for 12 h. Bacterial growth curves were plotted based on the average OD_600_ value. The growth rate (μ) was calculated from the OD_600_ data during the logarithmic growth phase (2–4 h) using the formula:


$$\mu =\frac{\ln\,\left({\mathrm{OD}}_{2}\right)-\ln\,\left({\mathrm{OD}}_{1}\right)}{t_{2}-t_{1}}$$

The area under the curve (AUC) of the growth curve was calculated using the trapezoidal rule with the formula:


$$\mathrm{AUC}=\sum_{i=1}^{n-1}\frac{\left({\mathrm{OD}}_{i}+{\mathrm{OD}}_{i+1}\right)}{2}\cdot \left(t_{i+1}-t_{i}\right)$$

All data are presented as mean ± standard deviation (*n* = 3). Statistical analysis was performed using GraphPad Prism software (version 8.3.0). Unpaired *t*-test was used to compare the growth rate and AUC between the two groups, and the significance level was set at *p* < 0.05.

### Mouse bloodstream infection model

To verify the pathogenicity of the SD8-1 strain and the effect of the pSD8-1-2 plasmid on tigecycline treatment, we constructed a bloodstream infection model in mice. All experiments were performed in accordance with the principles and procedures in the Guide for the Care and Use of Laboratory Animals of the National Institutes of Health and approved by the Experimental Animal Ethics Committee of Wenzhou Medical University. Male BALB/c mice aged 7 weeks were housed and had free access to food and water. Neutropenia was induced in mice by intraperitoneal injection of cyclophosphamide twice, the first dose was 150 mg/kg and the second dose was 100 mg/kg, injected 3 days and 1 day before infection, respectively. Each mouse was injected with 1 × 10^6^ CFU of SD8-1 and ΔSD8-1 (ΔpSD8-1-2) through the tail vein, with six mice in each group. Tigecycline 15 mg/kg was injected subcutaneously 2 h after infection, and tigecycline 7.5 mg/kg was injected subcutaneously every 12 h thereafter. All surviving mice were killed after 48 h, and liver, lung and kidney tissues were obtained for HE staining to observe tissue pathological changes.

### Whole-genome sequencing

Genomic DNA from a *tet*(X)-positive *R. ornithinolytica* strain was extracted using the TIANamp Bacteria DNA Kit (Tiangen, China). Sequencing was performed on the Illumina HiSeq 2500 platform (Bionova Biotech Co.), followed by *de novo* assembly using SPAdes v3.12.0. Antibiotic resistance genes were identified with ResFinder v3.1,^1^ while transposons and insertion sequence (IS) elements were detected using ISfinder.^2^ Additionally, integrative and conjugative elements (ICEs) were predicted with ICEberg 2.0 (Liu et al., 2019). Functional annotation was conducted using the NCBI Prokaryotic Genome Annotation Pipeline (PGAP) and RAST server. For strain SD8-1, long-read sequencing was performed using the Nanopore system, with assembly carried out using Unicycler v0.4.1. Plasmid collinearity was visualized and compared using Easyfig. The 545 *R. ornithinolytica* genome sequences analyzed in this study were retrieved from the NCBI database, with data collected up to 1 March 2025.

## Results

### Identification and antimicrobial susceptibility testing of *R. ornithinolytica* SD8-1

From June 2023 to December 2024, 38 *tet*(X)-positive bacterial strains were identified from 141 wastewater samples collected across eight pig farms by a swine wastewater resistance monitoring project in Shandong Province, China. These comprised 32 *E. coli*, 2 *Enterobacter cloacae*, 2 *Myroides* spp., 1 *Proteus mirabilis*, and 1 *R. ornithinolytica* (designated SD8-1) strains, which were verified via PCR and 16S rRNA gene sequencing. This study focused on SD8-1, and the other strains will be described in detail in subsequent studies. Antibiotic susceptibility testing revealed that SD8-1 exhibited resistance to tigecycline (MIC 4 mg/L), fosfomycin (> 256 mg/L), ampicillin (> 256 mg/L), and cephalosporins (e.g., ceftriaxone and cefotaxime). However, it remained susceptible to colistin, meropenem, and ciprofloxacin (Supplementary Table 1).

### Genomic analysis of *R. ornithinolytica* SD8-1

Whole-genome sequencing of SD8-1 was performed using short- and long-read approaches. The genome comprises a 5,489,588 bp chromosome and four plasmids: 78,159, 75,774, 33,222, and 10,755 bp. Average nucleotide identity analysis confirmed its identity as *R. ornithinolytica* (99.48% identity to *R. ornithinolytica* RoM27LC23, GCF_030505655.1). SD8-1 harbors multiple ARGs, including those conferring resistance to aminoglycosides [*aadA16*, *aac(6’)-Ib-cr*], β-lactam (*bla*_PLA_, *bla*_TEM–1B_), fosfomycin (*fosA*), chloramphenicol (*floR*, *catA2*), ciprofloxacin (*qnrB6*), rifamycin (*ARR-3*), and folate pathway antagonists (*sul1*, *dfrA27*), and tetracyclines [*tet*(X4), *tet*(D)]. Of these, *bla*_PLA_ and *fosA* are chromosomally encoded, whereas *tet*(X4) is located on a 78,159 bp IncFII(pCRY)-like plasmid (81% replicon similarity to pCRY), which was designated as pSD8-1-2. The remaining 11 resistance genes were found on a 75,774 bp multidrug-resistant IncR-IncFIA(HI1) hybrid plasmid, named pSD8-1-3 (Supplementary Table 2). The resistance phenotype of SD8-1 closely corresponds to its genotype. Additionally, the chromosome of SD8-1 encodes several virulence genes, including *fimH* (type 1 fimbriae D-mannose-specific adhesin, mediating bacterial adhesion), *fyuA* (siderophore yersiniabactin receptor, facilitating iron uptake), *irp2* (yersiniabactin non-ribosomal peptide synthetase, involved in iron acquisition), *mchF* (ABC transporter, aiding nutrient transport), and *nlpI* (lipoprotein, contributing to membrane stability), which collectively enhance adhesion, iron acquisition, and transport capabilities.

### Characterization of *tet*(X4)-bearing plasmid pSD8-1-2

**FIGURE 1:**
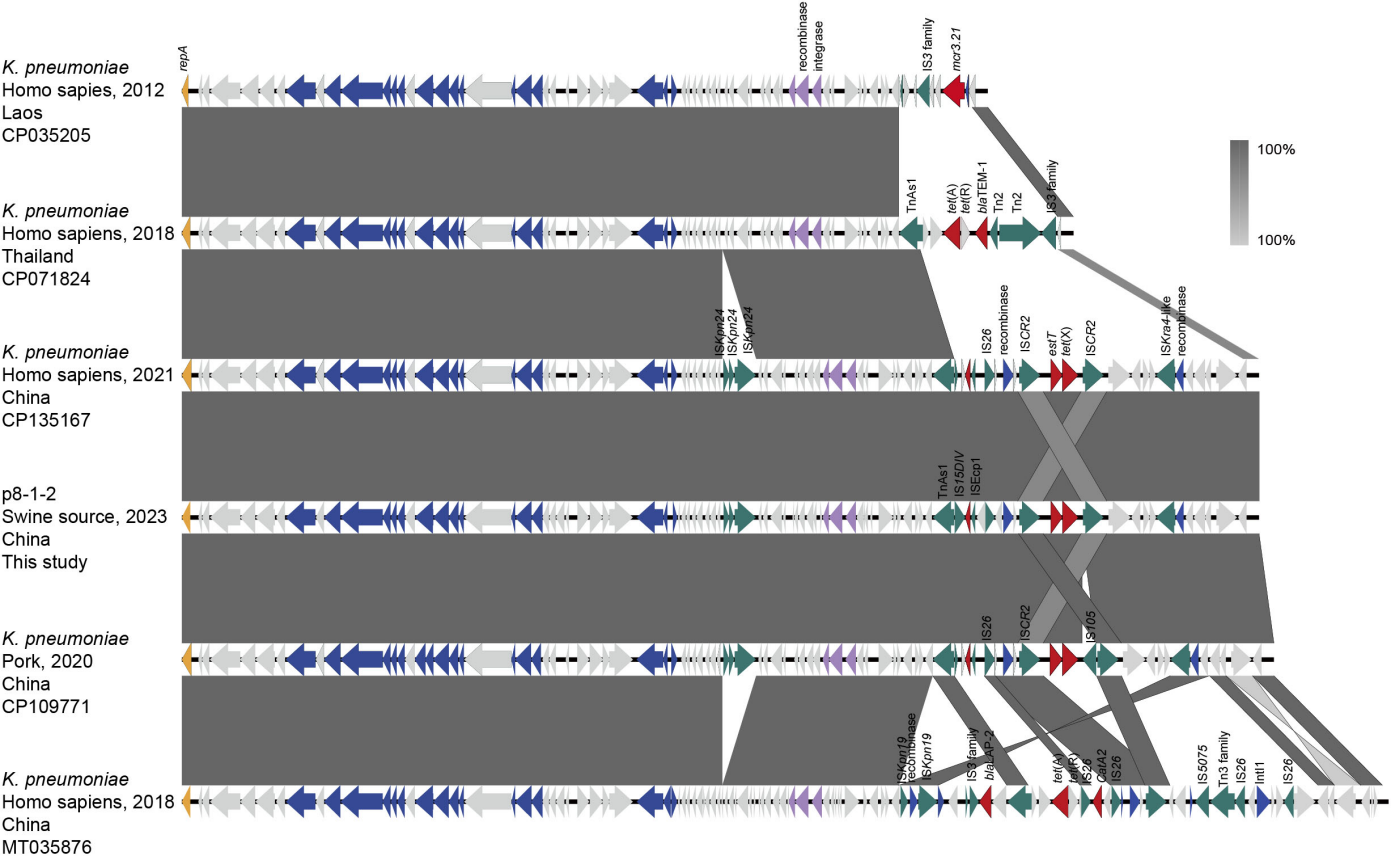
Linear sequence comparison of pSD8-1-2. Regions of homology are marked by shading. Arrows show the direction of transcription of open reading frames. The delta (Δ) symbol indicates a truncated gene. T4SS component, antimicrobial resistance, transposases, etc., are marked according to the colors in the legend to indicate their functions.

The IncFII(pCRY)-like plasmid pSD8-1-2, harboring *tet*(X4), is 78,159 bp-long with a GC content of 53%, and encodes 97 open reading frames. It comprises a 60.0 kb core region, harboring a type IV secretion system associated with plasmid conjugation, and an 18.2 kb variable region containing multiple insertion sequences (Figure 1). Based on searches on the National Center for Biotechnology Information (NCBI) database, pSD8-1-2 was found to share 100% query coverage and ≥ 99.99% identity with the *tet*(X)-positive plasmids in *Klebsiella* spp., including pNTT31XS-tetX4 (CP077430.1), pSDP9R-tetX4 (MW940621.1), pL3995-3 (CP135167.1), and pYZ-58-tetX (CP109771.1). Additionally, it exhibited 99.99% identity and 80%–90% coverage with *tet*(X4)-negative IncFII plasmids, with differences restricted to the variable regions. These IncFII-type plasmids typically carry 1–3 resistance genes, such as mcr-3.21, catA2, bla_*TEM–1*_, bla_*LAP–2*_, and *tet*(A), in their variable regions (Figure 1). A total of 84 pSD8-1-2-like plasmids and contigs (coverage > 80%) were identified, all within *Klebsiella* spp., with 80/84 in *K. pneumoniae*. The 18.2 kb variable region of pSD8-1-2, flanked by a recombinase gene upstream and Tn*AS1* downstream, contains an IS*kra4*-like element, IS*26*, and two IS*CR2* elements in the same orientation. Notably, *tet*(X4) resides within a conserved genetic cassette flanked by two IS*CR2* elements, a structure linked to the dissemination of *tet*(X) variants (Cui et al., 2020, 2022; Jin et al., 2024). Thus, pSD8-1-2 was likely formed via at least two recombination events: the assembly of the variable region and the IS*CR2*-mediated insertion of *tet*(X4).

**FIGURE 2:**
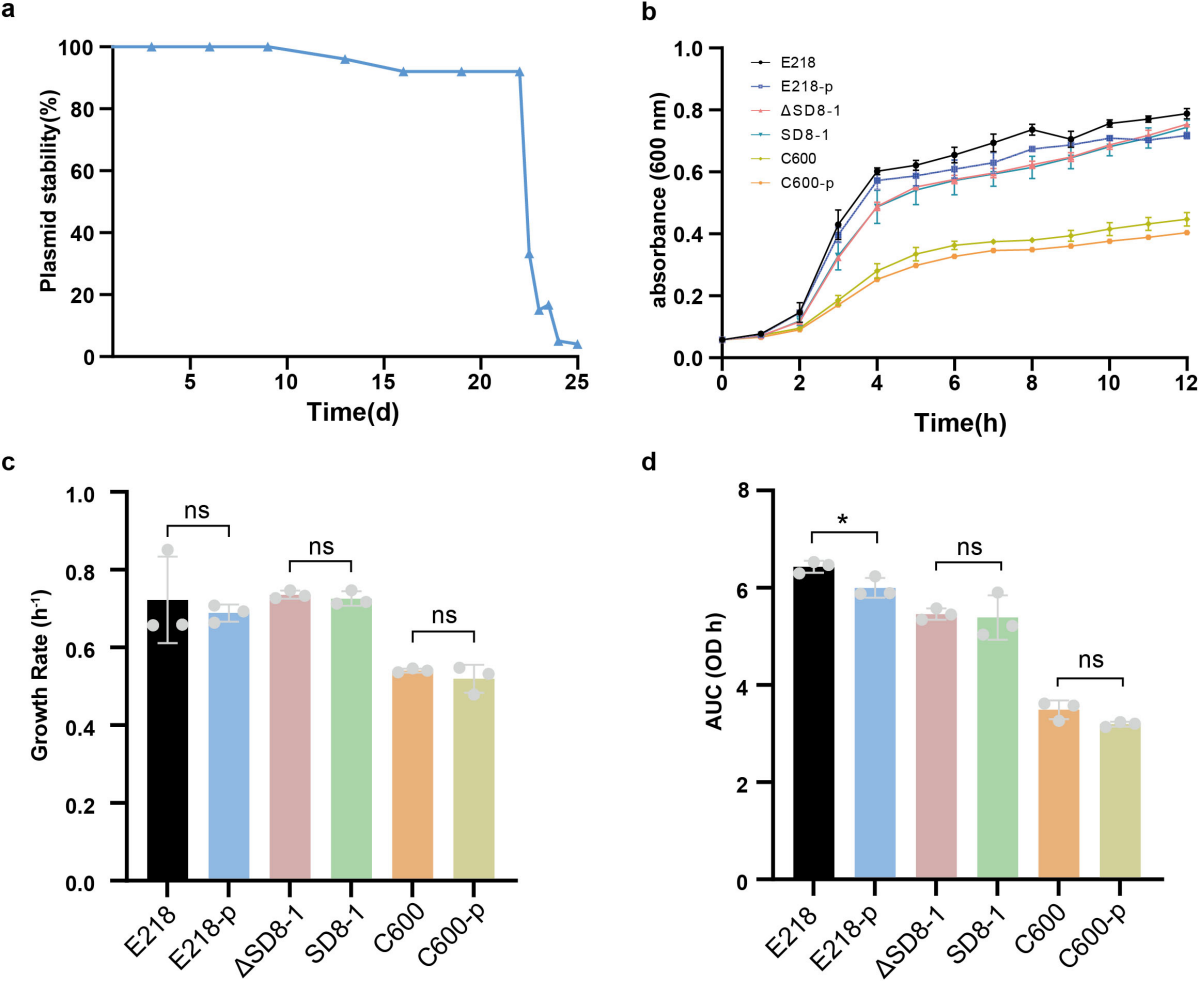
Analysis of bacterial growth and plasmid stability. **(a)** Plasmid stability of pSD8-1-2 in SD8-1, assessed by the retention rate of the *tet*(X4) gene over 25 days in antibiotic-free LB broth, with subculturing every 12 h and PCR confirmation of 50 colonies daily. **(b)** Growth curves of E218, E218-p, ΔSD8-1, SD8-1, C600, and C600-p strains, measured as OD_600_ over 12 h in LB broth at 37 °C. **(c)** Growth rates (μ) of the strains during the logarithmic phase (2–4 h). **(d)** Area under the curve (AUC) of the growth curves, computed using the trapezoidal rule. statistical significance was determined by unpaired *t*-test (*p < 0.05; ns, not significant).

Given the dissemination of pSD8-1-2 and related plasmids in *K. pneumoniae*, their stability and transferability were further investigated. Stability tests showed that pSD8-1-2 was fully retained in the host strain SD8-1 after 10 days of serial subculturing without selective pressure. Plasmid loss began after 13 days, with 8% loss by day 20, increasing to 84% by day 24 (Figure 2a). This indicates this plasmid is highly stable and can persist for an extended period in the absence of antibiotic selective pressure in SD8-1. The conjugation experiments demonstrated that the plasmid can spread across bacterial species, successfully transferring into *E. coli* C600 and the clinical *bla*_*NDM*_-producing pathogenic strain *E218*, with conjugation frequencies of 1.6 × 10^–5^ and 4.3 × 10^–6^, respectively. Despite multiple attempts, pSD8-2-1 could not be transferred into the *K. pneumoniae* strains O41 and O32. After acquiring pSD8-1-2, the minimum inhibitory concentration (MIC) of the recipient strains for tigecycline increased significantly by 32-fold. To assess the potential fitness cost of pSD8-1-2 to the host strains, growth curves were constructed for all tested strains, which showed that pSD8-1-2 did not significant affect the logarithmic phase growth rate of all tested strains (*E. coli* E218, *E. coli* C600, and *R. ornithinolytica* SD8-1) (*p* > 0.05) (Figures 2b, c). However, area under the curve (AUC) analysis revealed a statistically significant reduction in total growth yield for E218 (*p* < 0.05), while no significant effect was observed for SD8-1 and C600 (*p* = 0.831) (Figure 2d).

### Effect of pSD8-1-2 on *in vivo* tigecycline efficacy

**FIGURE 3:**
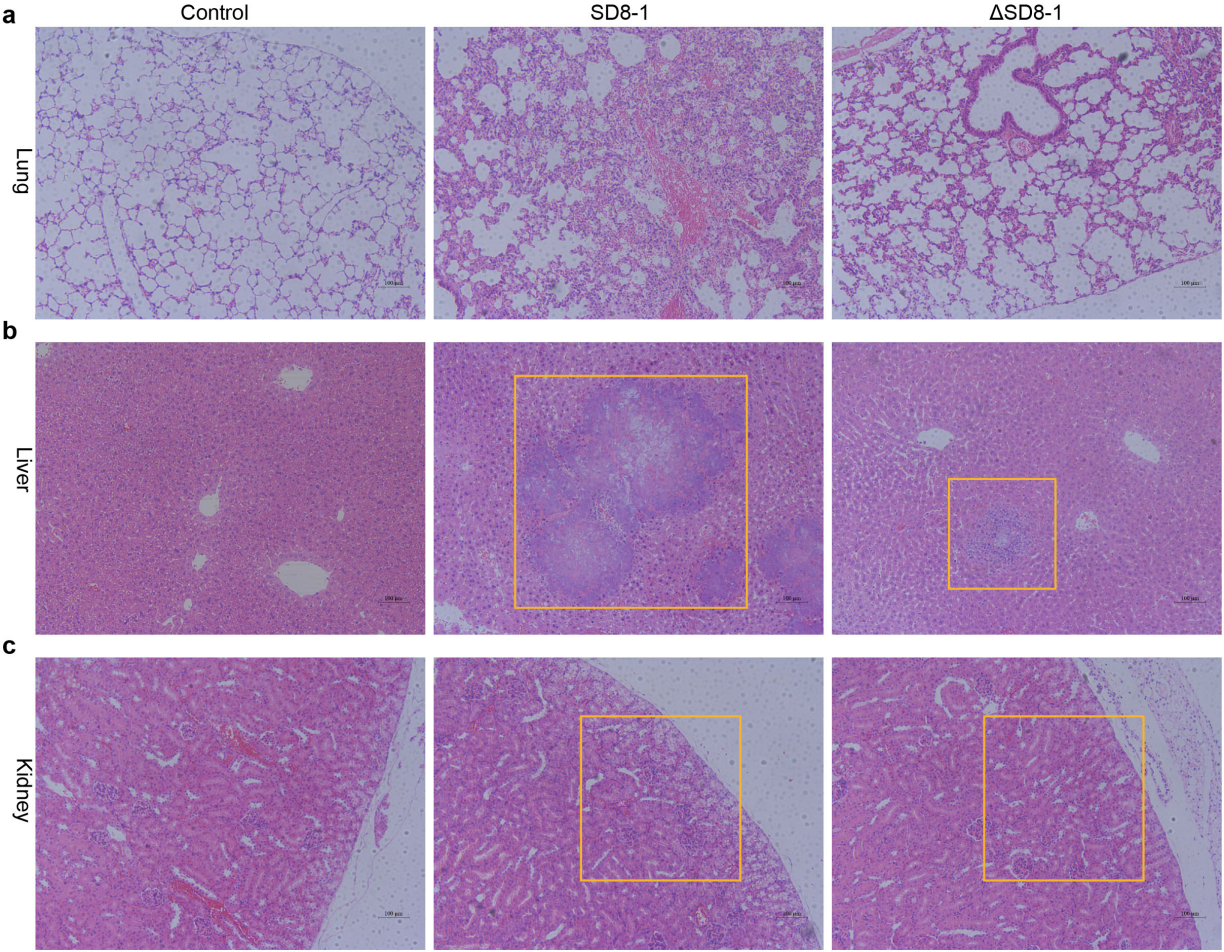
Histopathological analysis of mouse tissues in a bloodstream infection model with tigecycline treatment. **(a)** Lung tissue sections stained with hematoxylin and eosin (HE) from control, SD8-1, and ASD8-1 groups, showing alveolar structure and inflammatory changes. **(b)** Liver tissue sections from the same groups, highlighting hepatic architecture and potential lesions. **(c)** Kidney tissue sections, demonstrating renal histology and pathological alterations. Scale bar = 100 μm.

To assess the pathogenicity of *R. ornithinolytica* SD8-1 and the impact of pSD8-1-2 on the efficacy of tigecycline *in vivo*, a bloodstream infection model was established using BALB/c mice. Wild-type SD8-1 and plasmid-cured ΔSD8-1 strains (10^6^ CFU) were injected into the mice via the tail vein. Two hours’ post-inoculation, tigecycline was administered intraperitoneally daily for 2 days. During treatment, 50% of mice (3/6) in the wild-type group died, whereas all mice in the ΔSD8-1 group survived. After 2 days, the surviving mice were euthanized, and their lung, liver, and kidney tissues were collected for hematoxylin-eosin (H&E) staining to examine histopathological changes. In the wild-type group, the pulmonary tissues exhibited thickened alveolar septa, neutrophil and mononuclear cell infiltration, and proteinaceous exudates, indicative of acute inflammation. In contrast, the ΔSD8-1 group showed markedly reduced inflammatory cell infiltration and interstitial thickening, with partial restoration of alveolar architecture (Figure 3a). Hepatic tissues from the wild-type group displayed focal hepatocellular necrosis, Kupffer cell activation, and sinusoidal dilation with inflammatory cell infiltration. In contrast, the ΔSD8-1 group exhibited fewer Kupffer cells, reduced inflammatory infiltration, and partial restoration of liver lobule structure (Figure 3b). Similarly, tubular epithelial cell swelling, vacuolar degeneration, cell detachment, interstitial inflammation, and vascular congestion were observed in the renal tissues in the wild-type group; these changes were significantly alleviated in the ΔSD8-1 group, with minimal tubular injury, reduced inflammatory edema, and restored tubular lumens (Figure 3c). These survival and histopathological findings suggest that the *tet*(X4)-harboring pSD8-1-2 contributes to tigecycline treatment failure *in vivo*.

### Global distribution and resistance gene/replicon profiles of *R. ornithinolytica*

**FIGURE 4:**
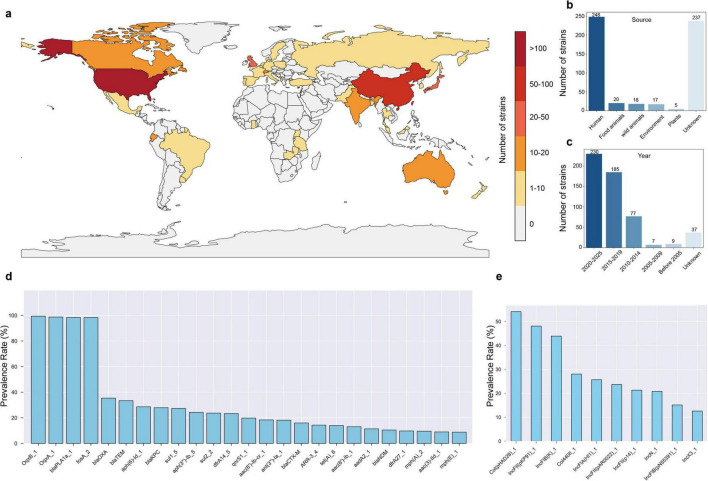
Global distribution and resistance profile of *R. ornithinolytica* strains. **(a)** World map showing the number of *R. ornithinolytica* strains isolated across 41 countries, colored by strain count (scale: 0 to > 100). **(b)** Source distribution of 545 strains. **(c)** Temporal distribution of genome submissions. **(d)** Carriage rates of the top 25 antibiotic resistance genes (ARGs). **(e)** Prevalence of the top 10 plasmid replicon types. Data are based on 545 non-duplicate genome sequences from the National Center for Biotechnology Information (NCBI) database, analyzed for antibiotic resistance genes and plasmid types.

To investigate the prevalence and distribution of antibiotic resistance in *R. ornithinolytica*, we analyzed 545 non-duplicate *R. ornithinolytica* genome sequences from the NCBI database, isolated from 41 countries across six continents. The majority originated from the United States (45.5%, 248/545), China (17.2%, 94/545), the United Kingdom (5.9%, 32/545), Japan (3.9%, 21/545), and Canada (2.9%, 16/545), with 18.3% (100/545) from other countries and 6.2% (34/545) from unspecified sources (Figure 4a). Most strains were isolated from humans (*n* = 228), followed by food animals (*n* = 20), wild animals (*n* = 18), and the environment (*n* = 17) (Figure 4b). Approximately 40% of the genome sequences (*n* = 230) were submitted between 2019 and 2024 (Figure 4c). Bioinformatic analysis revealed that four antibiotic resistance genes (*oqxA*, *oqxB*, *fosA*, *bla*_PLA_) were present in over 98% of *R. ornithinolytica* strains, conferring resistance to quinolones (*oqxAB*), fosfomycin (*fosA*), and β-lactams (*bla*_PLA_). The incidence rate of the 5*^th^* to 10*^th^* most common resistance genes, *bla*_*OXA*_, *bla*_*TEM*_, *aph(6)-Id*, *bla*_*KPC*_, *sul1*, *aph(3’)-Ia*, ranged from 24% to 35%, which conferred resistance to β-lactams, aminoglycosides, and sulfonamides (Figure 4d). Other resistance genes, with incidence rates from 8.81% (*mdfA*) to 23.67% (*dfrA14*), conferred resistance to multiple antibiotic classes, including quinolones (*qnrS1*) and trimethoprim (*dfrA14*). Additionally, *R. ornithinolytica* strains occasionally carried the colistin resistance gene *mcr* and the tigecycline RND-type efflux pump gene *tmexCD-toprJ*. Notably, 20% of strains exhibited MDR, carrying eight or more ARGs. Comparison of the plasmid replicon types between *R. ornithinolytica* and *K. pneumoniae* revealed that *R. ornithinolytica* strains predominantly carried Col(pHAD28), IncFII(pKP91), IncFIB(K), Col440II, IncFIA(HI1), IncFII(pAR0022), and IncFII(p14) replicons, with incidence rates ranging from 8% to 20% (Figure 4e). This result highlights a high prevalence of IncFII-type plasmids in *R. ornithinolytica* strains, underscoring their role in antibiotic resistance dissemination.

## Discussion

Livestock wastewater significantly contributes to the spread of resistant bacteria and ARGs from farms to the environment (Ibekwe et al., 2023; Zhu et al., 2020). Its abundant nutrients and residual antibiotics can increase the retention time of ARGs in the environment. The prevalence of ARGs, including those for β-lactamase (*bla*_CTX–M_), carbapenemases (*bla*_NDM_, *bla*_KPC_), and colistin resistance (*mcr*), is associated with the spread of their dominant plasmids (Bevan et al., 2017; Liu J. H. et al., 2024; Wu et al., 2019). Moreover, Alderliesten et al. (2020) found that conjugative plasmids can be transferred more efficiently between closely related species in a liquid environment. For example, compared with bacteria of the same species, plasmid conjugation frequency was reduced by about 0.37 times within the same family and 10 times within the same order (Alderliesten et al., 2020).

This study characterized the IncFII(pCRY)-like pSD8-1-2, carrying *tet*(X4), in *R. ornithinolytica* SD8-1. Using bioinformatic analysis, 84 pSD8-1-2-like plasmids or fragments were identified, predominantly in *K. pneumoniae* (80/84), across diverse ecological niches, including clinical human isolates, food animals, and meat products. These plasmids share a conserved backbone with pSD8-1-2, differing only in the variable regions harboring ARGs, such as *tet*(X4) within a conserved IS*CR2*-flanked cassette. Tigecycline is an important antibiotic for treating community- and hospital-acquired pneumonia, complicated intra-abdominal infections, and bloodstream infections caused by *K. pneumoniae*. It is typically used as a last-resort treatment for multidrug-resistant infections. Compared to its presence in bacteria like *E. coli*, the potential spread of *tet*(X4)-carrying pSD8-1-2 in *K. pneumoniae* poses a greater threat to clinical treatment, further limiting therapeutic options. The emergence of *tet*(X) genes in *R. ornithinolytica* suggests that the host range of *tet*(X) is expanding across species with the help of conjugative plasmids such as pSD8-1-2, which is exacerbated by the complex antibiotic environment in livestock wastewater.

pSD8-1-2 can transfer cross-species, successfully conjugating into *E. coli* C600 and clinical E218 with moderate efficiency (frequencies ranging from 1.6 × 10^–5^ to 4.3 × 10^–6^). Although the pSD8-1-2, with 100% coverage and 100% sequence identity, has been identified in the NCBI database (CP135167 and CP109771), our conjugation experiments failed to transfer pSD8-1-2 into the wild-type *K. pneumoniae* O41 and O32 strains, possibly due to interference from resident, incompatible IncFII-type plasmids in the recipient strains or the protective effects of cellular defense mechanisms such as restriction-modification and CRISPR–Cas systems (Siedentop et al., 2024; Womble et al., 1984). Stability assays revealed that pSD8-1-2 was retained in 92% of *R. ornithinolytica* SD8-1 after 20 days of serial passaging in an antibiotic-free environment. This high stability likely results from mechanisms such as partitioning systems, toxin-anti-toxin modules, and potential compensatory adaptations, which collectively ensure long-term plasmid maintenance. Notably, pSD8-1-2 imposed minimal metabolic burden, as it did not significantly affect the logarithmic growth phase rate across the tested strains (*E. coli* E218, *E. coli* C600, and *R. ornithinolytica* SD8-1) (*p* > 0.05). However, the total growth yield was slightly reduced in *E. coli* E218 (*p* < 0.05), suggesting a minor fitness cost. In contrast, *R. ornithinolytica* SD8-1 and *E. coli* C600 showed no significant reduction (*p* = 0.831), indicating that these strains were better adapted to hosting the plasmid, possibly due to optimized plasmid-host interactions. These results are consistent with the IncFII plasmid competition studies in *K. pneumoniae* (Li et al., 2021), underscoring the potential for widespread dissemination of pSD8-1-2 in diverse bacterial populations. Furthermore, this study confirmed the pathogenicity of *R. ornithinolytica* SD8-1 using an *in vivo* infection model and further demonstrated that pSD8-1-2 caused the failure of tigecycline treatment. Compared with the original SD8-1 group, the plasmid elimination group showed significant improvement in pathological signs.

In pSD8-1-2, *tet*(X4) is flanked by two 1,494-bp IS*CR2* insertion sequences in the same orientation, forming the conserved IS*CR2*-*aph*-*tet*(X4)-IS*CR2* structure. This structure, prevalent in *E. coli*, is capable of generating a ∼5 kb transposon intermediate that enhances *tet*(X4) mobility through horizontal gene transfer. IS*CR2*, the primary transposable element associated with *tet*(X), has been linked to all newly reported *tet*(X) variants, such as *tet*(X3) to *tet*(X7). Our recent studies have shown that in addition to assisting the spread of *tet*(X), IS*CR2* also contributes to the recombination between *tet*(X) variants, promoting the formation of several variants, including *tet*(X5) (Jin et al., 2024). In addition to *tet*(X), genomic analysis also revealed that IS*CR2* is associated with resistance to aminoglycosides, chloramphenicol, sulfonamides, and β-lactams (Che et al., 2021). These findings suggest that *tet*(X) may form an MDR cluster with these resistance genes through IS*CR2*, thereby creating favorable conditions for host bacteria to adapt to the complex antibiotic environment in farm wastewater.

Analysis of 545 non-duplicate *R. ornithinolytica* genome sequences from public databases highlighted their global prevalence and AMR profiles, providing insights into their epidemiological patterns and resistance mechanisms. These strains span 41 countries across six continents, with the United States (45.5%, *n* = 248) and China (17.2%, *n* = 94) being the primary contributors, likely reflecting differences in genome sequencing capacity, data submission frequency, and regional prevalence. The increased documentation of *R. ornithinolytica* strains from 2019 to 2024 (*n* = 230) corresponds with the advancements in sequencing technologies and enhanced data sharing. Host analysis revealed that humans are the primary source (*n* = 228), followed by food animals (*n* = 20), wild animals (*n* = 18), and environmental samples (*n* = 17), indicating *R. ornithinolytica* can transmit across the human-animal-environment interface, consistent with the “One Health” framework. The presence of environmental isolates suggests that the environment may serve as a reservoir and pathway for antibiotic-resistant bacteria, necessitating further investigation into its potential contribution to the transmission of ARGs. Multidrug-resistant phenotypes were also discovered in *R. ornithinolytica*, with high prevalence of carbapenem resistance genes, including *bla*_KPC_ (*n* = 152, 27.9%) and *bla*_OXA_ (*n* = 193, 35.4%), suggesting reduced efficacy of carbapenems. Additionally, *R. ornithinolytica* and *K. pneumoniae* were found to share highly prevalent plasmid replicon types, specifically IncFII(pKP91) and IncFIB(K), which are highly prevalent in both species (ranging from 40% to 50% in both) (Wyres et al., 2020). This shared replicon profile suggests that cross-species dissemination of resistance plasmids might occur between these two bacterial species.

Currently, the low prevalence of *tet*(X) genes in *K. pneumoniae* presents a critical window to curb their spread among clinically significant pathogens, such as carbapenem-resistant *K. pneumoniae* (CRKP) and hypervirulent *K. pneumoniae* (hvKP). Adopting a “One Health” approach that integrates human, animal, and environmental health, alongside coordinated cross-sectoral strategies, can significantly help mitigate this risk. In 2020, China banned the use of antibiotic growth promoters in animal feed (Ministry of Agriculture and Rural Affairs of the People’s Republic of China, 2020), markedly reducing the transmission of animal-derived antibiotic resistance to humans, representing a notable step forward in resistance control. However, continued use of tetracyclines, which remain the primary treatment options for bacterial infections in animal husbandry due to their affordability and broad-spectrum efficacy, may exert selection pressure. This might promote the spread of resistance genes, including *tet*(X) (Aminov, 2021), particularly through shared resistance plasmids between food animals and clinical isolates. Consequently, stricter regulations should be implemented on the non-essential use of older tetracyclines in agriculture to preserve the efficacy of tigecycline and next-generation tetracyclines, such as eravacycline and omadacycline. Furthermore, enhancing molecular surveillance of resistance genes, refining antibiotic stewardship guidelines, and promoting international collaboration in antibiotic resistance management can significantly benefit effective control (De Oliveira et al., 2020). These combined efforts are necessary to limit the spread of *tet*(X) and support the long-term sustainability of antibiotic therapies.

## Data Availability

All genomes have been deposited in GenBank under the BioProject PRJNA1255831.

## Footnotes

1
https://genepi.food.dtu.dk/resfinder

2
https://www-is.biotoul.fr
