# Supplementary material for: Emergence of tigecycline-resistant Raoultella ornithinolytica with tet(X)-carrying plasmid from swine wastewater in China
Source: Front Microbiol. 2025 Sep 16;16:1642708. doi: 10.3389/fmicb.2025.1642708 (PMC12479518; doi:10.3389/fmicb.2025.1642708)
Supplement: Supplementary file 1 [file Table_1.docx]

**Supplementary Information**

| Strain/drug | GEN | AMK | S/T | FFC | RIF | FOS | AMP | CTX | MEM | CS | CIP | TET | MIN | TGC |
| --- | --- | --- | --- | --- | --- | --- | --- | --- | --- | --- | --- | --- | --- | --- |
| SD8-1 | 0.5 | 1 | >320 | >256 | 128 | 128 | >256 | 0.25 | 0.03 | 0.25 | 4 | >256 | 64 | 8 |
| C600 | 0.5 | 1 | ＜5 | 2 | 8 | 2 | 2 | 0.125 | 0.03 | 0.25 | 2 | 2 | 0.5 | 0.03 |
| +pSD8-1-2 | 0.5 | 1 | ＜5 | 2 | 8 | 2 | 4 | 0.125 | 0.03 | 0.25 | 2 | 128 | 16 | 4 |

**Table S1. MICs of 14 antibiotics for the studied strains.**

GEN, gentamicin; AMK, amikacin; S/T, trimethoprim/sulfamethoxazole; FFC, florfenicol; RIF, rifampicin; FOS, fosfomycin; AMP, ampicillin; CTX, cefotaxime; MEM, meropenem; CS, colistin; CIP, Ciprofloxacin; MIN, minocycline; TGC, tigecycline;

**Table S2** **The Genetic Features of *Raoultella ornithinolytica* SD8-1**

|  | Size(kb) | Inc-type | Resistance genes |
| --- | --- | --- | --- |
| chromosome | 5,489.6 | - | *bla*_PLA1a_, *fosA* |
| pSD8-1-2 | 78.2 | IncFII(pCRY) | *tet*(X4) |
| pSD8-1-3 | 75.8 | IncFIA(HI1)-IncR | *aadA16, aac(6')-Ib-cr, bla*_TEM-1B_*, floR, catA2, qnrB6, ARR-3, sul1, tet*(D)*, dfrA27* |
| pSD8-1-4 | 33.2 | repB(R1701) | *bla*_TEM-1B_ |
| pSD8-1-5 | 10.8 | Col440I | - |
